# IL-33 and IL-3 synergistically induce CD25 expression on human basophils without functional IL-2 signaling: a potential marker of severe COVID-19

**DOI:** 10.3389/fimmu.2025.1718240

**Published:** 2025-12-11

**Authors:** Sruthi Vijaya Retnakumar, Suraj Chandrabhan Singh, Camille Chauvin, Jagadeesh Bayry

**Affiliations:** 1 Institut National de la Santé et de la Recherche Médicale, Centre de Recherche des Cordeliers, Sorbonne Université, Université Paris Cité, Paris, France; 2 Department of Biological Sciences & Engineering, Indian Institute of Technology Palakkad, Palakkad, India

**Keywords:** basophils, CD25, IL-2, IL-3, IL-33, Treg, COVID-19, Type 2 immunity

## Abstract

CD25 (IL-2 receptor alpha chain) is a key component of the high-affinity receptor for the IL-2 cytokine and regulates the proliferation, survival, and differentiation of immune cells. The expression of CD25 has been demonstrated in basophils of patients with allergic diseases such as asthma. Previous studies have shown that IL-3 priming leads to increased CD25 expression in basophils. However, other potential stimuli that can regulate the CD25 expression on basophils and their possible biological consequences remain poorly understood. Here, we demonstrate that the IL-33 cytokine, a critical driver of type 2 immunity and allergic inflammation, significantly upregulates CD25 expression on IL-3-primed human basophils. Despite high levels of CD25 expression and its ability to capture IL-2 cytokine, basophils fail to express CD122 (IL-2 receptor beta chain), resulting in the absence of downstream signal transducer and activator of transcription 5 (STAT5) phosphorylation and lack of IL-2-mediated survival signals. The binding of IL-2 to basophil-derived CD25 appears to be transient since co-culturing with regulatory T cells (Treg cells) demonstrates that IL-2 remains accessible to Treg cells, enhancing their viability. Furthermore, an analysis of publicly available single-cell RNA sequencing data from COVID-19 patients revealed increased gene expressions of *CD25* and *CD132* but not *CD122* in basophils from severe patients, which is associated with higher levels of *IL-3* and *IL-33*, positioning CD25 on basophils as one of the potential biomarkers for severe COVID-19. Our data advance the understanding of basophil biology and highlight the complex regulation of IL-2 receptor components in basophil-driven inflammation.

## 1 Introduction

Basophils are a rare subset of granulocytes that constitute less than 1% of circulating white blood cells and play essential roles in type 2 immune responses against allergens and parasites. They are typically characterized by the expression of the high-affinity immunoglobulin (Ig) E receptor Fc epsilon Receptor I (FcϵRI) and the interleukin (IL)-3 cytokine receptor cluster of differentiation (CD)123. Basophil activation commonly occurs through crosslinking of IgE bound to FcϵRI, triggering rapid degranulation and release of preformed mediators such as histamine and leukotrienes, alongside the synthesis of cytokines including IL-4 and IL-13 (1). However, basophils can also be regulated and activated through IgE-independent mechanisms, especially cytokines such as IL-3, granulocyte-macrophage colony-stimulating factor (GM-CSF), IL-5, and IL-33 and signaling via pattern recognition molecules (2).

IL-2 is a pleiotropic cytokine, which is primarily secreted by activated T cells and, to some extent, by monocytes and dendritic cells. IL-2 exerts its biological effects through a trimeric receptor complex consisting of alpha (α or CD25), beta (β or CD122), and gamma (γ or CD132) chains. The CD25 subunit is unique to IL-2 binding, while CD122 is shared with IL-15, and CD132 is common to all IL-2 family cytokines. CD25 alone can bind IL-2 with low affinity; nevertheless, it does not trigger intracellular signaling. In contrast, the beta and gamma chains (CD122 and CD132) assemble into a dimeric, intermediate-affinity functional receptor (IL-2Rβ/γ_c_ complex), which is constitutively present on natural killer (NK) cells and resting lymphocytes (3). Upon lymphocyte activation, cells transiently upregulate CD25, assembling the high-affinity trimeric receptor—resulting in approximately 1000-fold increased affinity for IL-2 and robust intracellular signaling. Certain cell populations, such as regulatory T cells (Treg cells) and type 2 innate lymphoid cells (ILC2), constitutively express this high-affinity trimeric IL-2 receptor, allowing them heightened sensitivity to IL-2 in the immune microenvironment (4).

Although normal resting basophils have minimal expression of CD25 (IL-2Rα), there have been previous reports demonstrating that long-term priming with IL-3 upregulates CD25 receptor expression on the surface of basophils (5, 6) and mast cells (7). This upregulation is also observed in basophils from patients with allergic asthma and peanut allergy compared to healthy individuals, suggesting a disease-associated priming state (8, 9). Despite these observations, the regulation of CD25 expression by other basophil mediators and the possible downstream signaling events and functional consequences of CD25 upregulation on basophils have not been investigated so far. Moreover, the relevance of basophils and the cytokines IL-3 and IL-33 extends beyond allergic disorders into a broad spectrum of disorders, including coronavirus disease of 2019 (COVID-19), tissue fibrosis, and autoimmune diseases (10, 11), suggesting additional contexts in which CD25 regulation on basophils warrants further investigation.

In this study, we investigated the effects of various cytokines on CD25 expression in purified human basophils, with and without IL-3 priming. We found that IL-33 strongly enhances CD25 expression and promotes its shedding as soluble form (sCD25) in IL-3-primed basophils. Although these CD25-expressing basophils effectively bind IL-2, engagement by IL-2 did not induce either canonical downstream signaling or affect their survival due to the basal level expression of functional intermediate (IL-2Rβ/γ_c_ complex), or high-affinity receptor forms (IL-2Rαβ/γ_c_), which are required for transduction of IL-2 signals. Moreover, co-culture experiments with Treg cells revealed that basophil-derived CD25 does not sequester IL-2 from Treg cells; instead, IL-2–bound basophils preserve IL-2 availability and consequently support Treg cell viability. In parallel, we observed a similar expression pattern of IL-2 receptors in COVID-19 patients, where basophils from severe cases showed upregulation of *CD25* and *CD132*, but not *CD122*, a profile that correlated with elevated levels of *IL-3* and *IL-33*. Together, these findings uncover new aspects of basophil regulation and raise important questions about the role of IL-2 sequestration and release by basophils in modulating responses of other IL-2-dependent immune cells in allergic and inflammatory pathologies.

## 2 Materials and methods

### 2.1 Reagents and antibodies

The following fluorochrome-conjugated antibodies were used for flow cytometry staining. CD25-fluorescein isothiocyanate (FITC) (clone: M-A25, Cat. Cat no. 356106), CD69-allophycocyanin (APC)/Cy7 (clone: FN50), CD203c-APC (clone: E-NPP3, Cat no. 324610), phosphorylated signal transducer and activator of transcription 5 (pSTAT5)-alexa fluor (AF)488 (clone: 47/Stat5(pY694), Cat. Cat no. 612598), CD56 (NCAM-1)-AF700 (clone: B159, Cat no. 557919), Propidium Iodide (PI) staining solution (Cat no. 556463) were from BD Biosciences. CD25-phycoerythrin (PE) (clone: M-A251, Cat no. 356104), FcϵRIα Brilliant Violet 421 (clone: AER-37/CRA-1, Cat no. 334624), CD122-APC (clone: TU27, Cat no. 339008), CD132-PE (clone: TUGh4, Cat no. 338606), and CD4-Peridinin-Chlorophyll-Protein (PerCP) Cyanine 5.5 (clone: SK3, Cat no. 344608) from BioLegend. FcϵRIα-APC (clone: CRA1, Cat no. 130-095-980) was from Miltenyi. Annexin V-APC (Cat no. BMS306APC), fixable viability dye eFluor 506 (Cat no. 65-0866-14) were from eBioscience. Streptavidin-AF488 conjugate (Cat no. S32354) was from Invitrogen.

Recombinant human IL-3 was purchased from ImmuCat no. ools (Cat no. 11340037, Friesoythe, Germany). IL-33 was from Peprotech (Thermofisher Scientific), and IL-2 was from Miltenyi Biotech (Cat no. 130-097-743). Biotinylated IL-2 was purchased from R&D Systems (Cat no. BT202-025/CF).

### 2.2 Isolation of basophils and Treg cells

Buffy coats of healthy donors were obtained from Etablissement Français du Sang, Rungis, France (Institut National de la Santé et de la Recherche-EFS ethical committee convention 18/EFS/033) and were subjected to Ficoll density gradient centrifugation to separate the cellular fractions containing peripheral blood mononuclear cells (PBMCs) and basophils. Basophils were negatively isolated from these fractions using basophil isolation kit II (Miltenyi Biotec). CD4^+^CD25^+^CD127^dim/–^ regulatory T Cell Isolation Kit II (Miltenyi Biotec) was used for the isolation of CD4^+^CD25^+^CD127^dim/–^ Treg cells from human PBMCs.

### 2.3 Culturing of basophils

Freshly isolated basophils were plated in a 96-well plate at a concentration of 0.1 million/200 μl/well in serum-free X-VIVO 15 media (Lonza). They were either left alone or primed with IL-3 (10 ng/ml) for 1 hr, followed by treatment with IL-33 (5 ng/mL) for 24 hrs, at 37 °C, 5% CO_2_, unless otherwise indicated in the legend. After incubation, the supernatants were collected and stored for cytokine analysis and the cells were processed for surface staining of various markers, such as FcϵRIα, CD25, CD122, and CD132, by flow cytometry. The cells were fixed with 4% paraformaldehyde and were acquired using LSR II (BD Biosciences). The data were analyzed by FlowJo software.

### 2.4 Enzyme-linked immunosorbent assay

Cell-free supernatants from basophils were analyzed for soluble CD25 (sCD25) using human CD25/IL-2R alpha quantikine ELISA kit from Biotechne, R&D systems. The cytokines IL-4 and IL-13 were measured using uncoated ELISA kits (Invitrogen) according to the manufacturer’s protocol.

### 2.5 IL-2 binding assay

After treatment with IL-3 and IL-33 for 24 hrs, basophils were harvested and washed with 1X Phosphate-Buffered Saline (PBS). Human biotinylated-IL-2 was added to the cells at a concentration of 5 μg/mL in 100 μL PBS and incubated for 30 min at room temperature. Cells were washed twice with PBS and extracellularly bound IL-2 was detected with streptavidin-AF488 by flow cytometry.

### 2.6 Evaluation of STAT5 phosphorylation by intracellular staining

Basophils were treated alone or in the presence of IL-3 and IL-33 for 24 hrs to induce the expression of CD25. The cells were washed 3 times with 1 X PBS to remove the cytokines and recombinant human IL-2 was added at 3000 U/ml for 30 min at 37 °C and 5% CO_2_. The cells were then harvested and processed for intracellular staining of pSTAT5 using Cell Signaling Buffer Set A (Miltenyi Biotec, Cat. no. 130-100-827) as per the manufacturer’s instructions followed by acquisition with flow cytometer.

### 2.7 Measurement of apoptosis by annexin-PI staining

Basophils were treated alone or in the presence of IL-3 and IL-33 for 24 hrs to induce the expression of CD25. IL-2 was added to the culture at the indicated concentrations for another 24 hrs. The cells were collected and stained with Annexin V and PI, followed by immediate acquisition with flow cytometer.

For co-culture experiments with Treg cells, basophils were cultured in the presence of IL-3 and IL-33 for 72 hrs. During last 24 hrs, IL-2 was added to the culture at 20 U/mL. Subsequently, isolated Treg cells were introduced to the culture at a 1:1 basophil: Treg ratio and co-cultured for an additional 48 hrs. As a positive control, Treg cells alone were treated with IL-2 under identical conditions. Treg cells were identified by gating on CD4 and CD25 surface markers, and apoptosis was evaluated using Annexin V and PI staining.

### 2.8 Data sources

We analysed publicly available single-cell RNA sequencing (scRNA-seq) datasets of bronchoalveolar lavage fluid (BALF) from two independent cohorts of patients with COVID-19 downloaded from NCBI GEO database. The first cohort comprised 6 severe and 3 moderate COVID-19 patients and 3 healthy control (accession number GSE145926) (12), and the second cohort included 4 COVID-19 patients (accession number GSE149878) (13). To increase the number of control cells and improve the robustness of comparisons, additional scRNA-seq data from BALF from one additional healthy control were acquired from the Gene Expression Omnibus (GEO) database under accession code GSE128033 (14), which contains data of one fresh BALF (GSM3660650) from a lung transplant donor.

### 2.9 Data processing and integration

Raw gene expression matrices were processed using the Seurat R package (v5.3.0) (15). Cells with fewer than 200 detected genes, >10% mitochondrial gene content, and >5% ribosomal gene content were excluded. Genes expressed in fewer than 3 cells were also removed. After filtering, gene expression counts were log-normalized and scaled separately for each sample. To correct for donor and batch-specific effects, datasets were integrated using the FindIntegrationAnchors and IntegrateData functions in Seurat with default parameters.

### 2.10 Cell type annotation

Cell identities were assigned using the SingleR package (v2.8.0) (16), using MonacoImmuneData as a reference dataset, and transcriptomics profiles of each cell were compared with this reference to annotate each cell with a different immune cell type. Basophils were extracted from the integrated object for downstream analysis. Basophils were annotated by using a set of genes “*IL3RA*”, “*FCER1A*”, “*MS4A2*”, “*HDC*”, “*CPA3*”, “*CLC*”, “*GATA2*”, “*IL5RA*”, “*ENPP3*”, “*CD200R1*”, “*GCSAML*”, “*PTGDR2*”. Treg cells were annotated by a set of genes “*FOXP3*”, “*IL2RA*”, “*CTLA4*”, “*IKZF2*”, “*TNFRSF18*”, “*LAG3*”, “*TIGIT*”, “*CCR4*”.

### 2.11 Gene expression analysis

Global expression of *IL3* and *IL33* was assessed in the integrated dataset across the groups. In the basophil subset, expression of *IL2RA* (CD25), *IL2RB* (CD122), and *IL2RG* (CD132) was evaluated across COVID-19 patients and control groups. Normalized expression distributions between COVID-19 patients (mild and severe) and healthy controls were compared using the Wilcoxon rank-sum test. Violin plots were generated with ggplot2 to visualize group differences.

### 2.12 Statistical Analysis

Statistical analyses of flow cytometry data were performed by one-way Analysis of Variance (ANOVA) with Tukey’s multiple comparisons post-test as indicated in the figure legends using Prism 8 (GraphPad Software Inc, CA). P < 0.05 was considered significant.

## 3 Results

### 3.1 IL-33 upregulates CD25 expression in IL-3 primed human basophils

**Figure 1:**
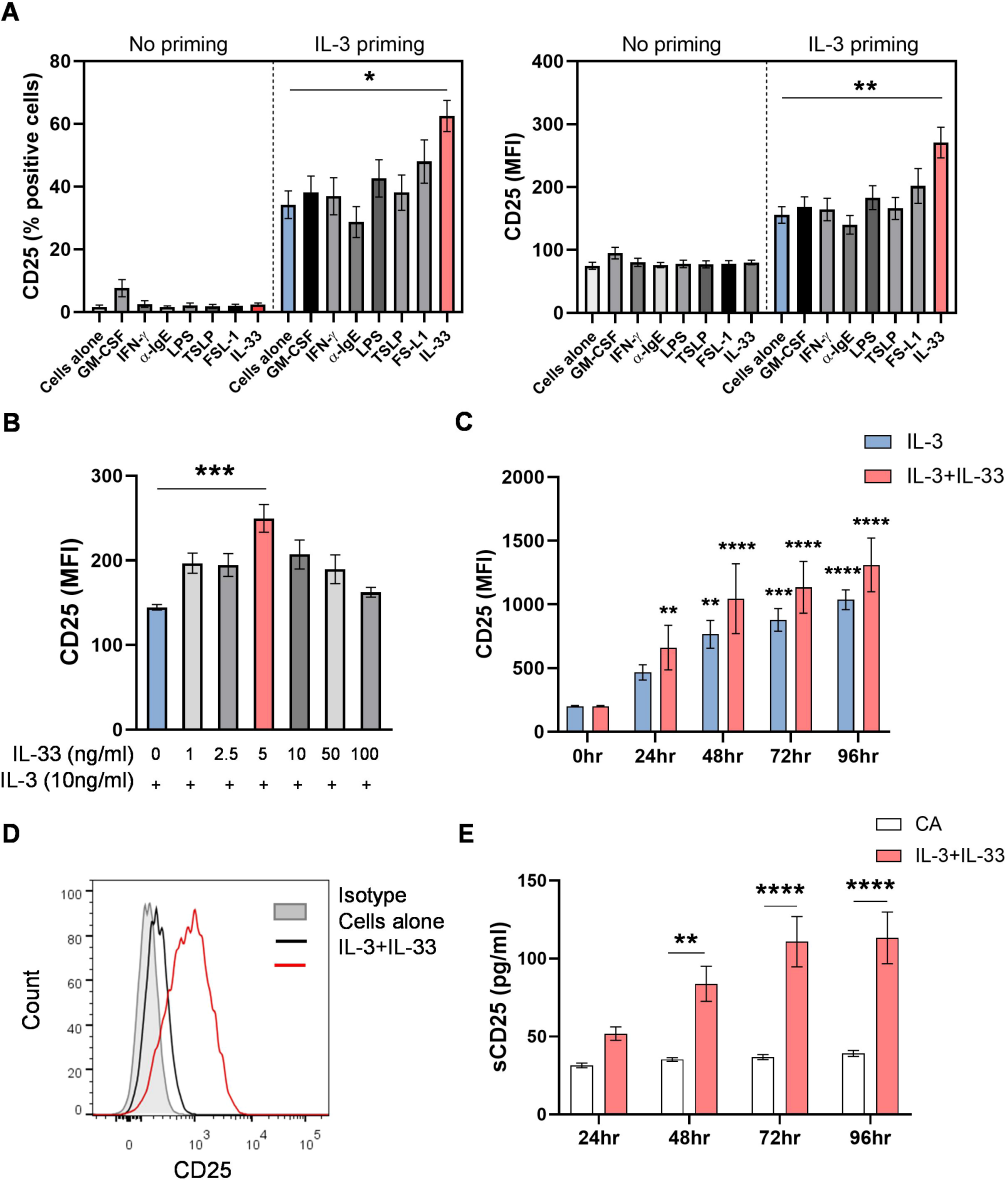
IL-33 upregulates IL-2 receptor alpha chain (CD25) expression in IL-3 primed human basophils. Basophils isolated from PBMCs of healthy donors were cultured alone or in the presence of various stimuli with or without IL-3 (10 ng/ml) priming. **(A)** Each of the indicated stimulatory agents was added either alone or after 1 hr of IL-3 priming, and CD25 expression was evaluated by FACS after 24 hrs. Summarized data (% positive cells and MFI) for n = 6 independent donors. **(B)** Dose response of IL-33 cytokine on the expression of CD25 (MFI, n = 3). **(C)** Time kinetics of IL-3 and IL-33 on the expression of CD25 up to 96 hrs compared to time 0. IL-33 was added at 5 ng/ml after 1 hr of priming with IL-3 (10 ng/ml). The culture was supplemented every 24 hrs with additional IL-33 (5 ng/ml) (MFI, n = 3). **(D)** Representative FACS histogram for CD25 expression on basophils compared to isotype control. **(E)** Levels of soluble CD25 in the culture supernatants of basophils either cultured alone or stimulated with IL-3 and IL-33 at different time points (n = 6). Data are represented as mean ± SEM. *P < 0.05, **P < 0.01, ***P < 0.001, ****P < 0.0001; ns, not significant by one-way ANOVA **(A, B)** or 2-way ANOVA **(C, E)** followed by Tukey’s multiple comparison test. Abbreviation: MFI, median fluorescence intensity.

To investigate the modulation of IL-2 receptor expression on primary human basophils, we have screened for various potential stimulatory agents for their ability to induce CD25 *in vitro*. The basophils isolated from healthy donors were either cultured alone or primed with IL-3 (10 ng/ml) for 1 hr, followed by treatment with the indicated stimuli for 24 hrs, and the surface expression of CD25 was measured by flow cytometry. While no expression of CD25 was detected with any of the stimuli without IL-3 priming, IL-3 alone partially induced the expression of CD25 as previously reported (5, 6), and treatment with IL-33 cytokine in combination with IL-3 significantly enhanced the expression of CD25 among other stimulatory agents (**Figure 1A**). However, classical basophil activation markers such as CD69 and CD203 were unaffected by IL-33 addition. (**Supplementary Figure S1A**). A dose-response relationship analysis of IL-33 cytokine and CD25 expression on primed basophils showed maximum CD25 levels at 5 ng/ml (**Figure 1B**). Furthermore, a time-kinetics analysis of CD25 expression over 96 hrs revealed sustained upregulation when basophils were initially stimulated with IL-3 and IL-33, followed by repeated IL-33 supplementation every 24 hrs (**Figure 1C**). **Figure 1D** shows the representative histogram overlay of CD25 expression on unstimulated and IL-3 + IL-33-stimulated basophils compared to the isotype control. Since CD25 is known to be shed in soluble form from CD25^+ve^ cells, we have also measured sCD25 in cell-free supernatants at different timepoints. As shown in **Figure 1E**, continuous exposure of basophils to IL-3 and IL-33 resulted in significant upregulation of sCD25, which closely correlated with surface CD25 expression.

IL-33 is a tissue-derived alarmin cytokine critically involved in type 2 innate immunity and allergic immune responses, which signals through classical myeloid differentiation primary response 88 (MyD88)/interleukin-1 receptor-associated kinase 1 (IRAK1)/tumor necrosis factor receptor-associated factor 6 (TRAF6) pathway upon binding to its receptor ST2 (17, 18). Although freshly isolated basophils do not express detectable levels of ST2 receptor, it is inducible by IL-3 priming. Consequently, in line with previous reports (19, 20), IL-33 synergistically enhanced cytokine production in IL-3–primed basophils (**Supplementary Figure S1B**).

### 3.2 Expression of IL-2 receptor alpha (CD25), beta (CD122) and gamma (CD132) chains on basophils

**Figure 2:**
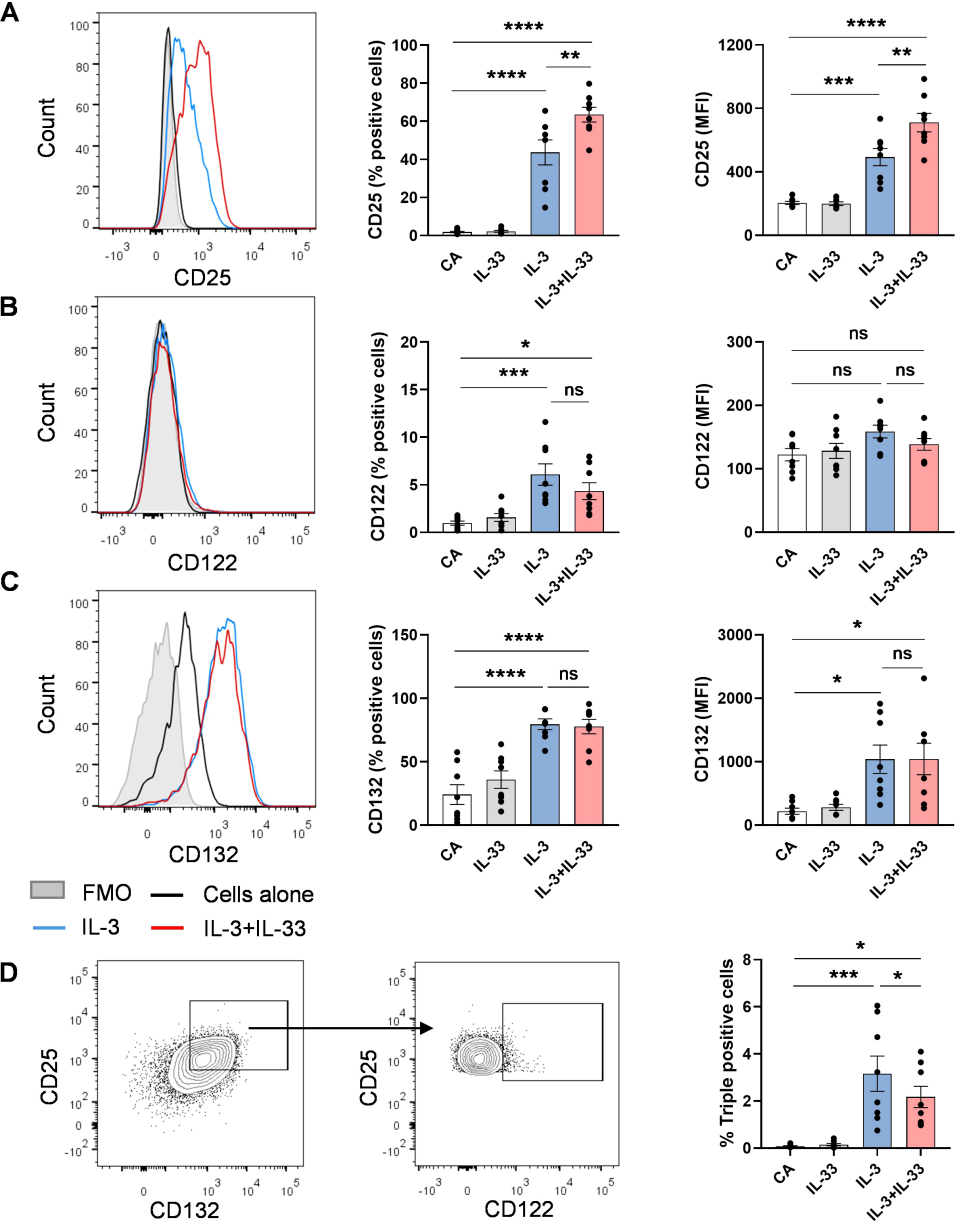
Expression of IL-2 receptor alpha (CD25), beta (CD122) and gamma (CD132) chains on IL-3 + IL-33 stimulated basophils. CD25 (IL-2Rα), CD122 (IL-2Rβ), and CD132 (IL-2Rγ) expression was analyzed on basophils after 24 hrs of stimulation with or without IL-3 and IL-33 cytokines as indicated. **(A-C)** Representative FACS histogram and summarized data for the expression level of CD25, CD122, and CD132 (% positive cells and MFI). **(D)** Percentage of cells expressing the trimeric IL-2 receptor was identified by successive gating on each of the receptor chains. Data are represented as mean ± SEM (n = 8). *P < 0.05, **P < 0.01, ***P < 0.001, and ****P < 0.0001; ns, not significant by one-way ANOVA followed by Tukey’s multiple comparison test. Abbreviation: CA, cells alone; MFI, median fluorescence intensity.

Since the functionality of the IL-2R is dependent on the beta and gamma chains, we have investigated the expression of these subunits in response to IL-3 and IL-33 stimulation. **Figure 2** represents the co-staining of alpha, beta, and gamma chains of IL-2 receptor on basophils in response to stimulation with IL-3 and IL-33. While stimulation with IL-3 alone significantly induced the surface expression of CD25 and CD132 on up to 40% and 80% of the cells, respectively, the expression of CD122 was limited to 6% of basophils and the resulting median fluorescence intensity (MFI) of the total population was not significantly affected. The addition of IL-33 in combination with IL-3 further enhanced the expression of CD25, and the expression of CD122 and CD132 remained unchanged compared to IL-3 alone (**Figures 2A–C**). The percentage of cells expressing the trimeric IL-2R in response to the stimuli was only 2-3% of the total basophils (**Figure 2D**). The expression of different subunits was confirmed with the same antibodies on classical IL-2 responsive cell types, such as Treg cells (high-affinity trimeric IL-2 receptor) and CD56^+ve^ NK cells (intermediate-affinity dimeric receptor), as a positive control (**Supplementary Figure S2**).

### 3.3 Binding of IL-2 to CD25-expressing basophils neither induces downstream signaling nor limits IL-2 availability to Treg cells

**Figure 3:**
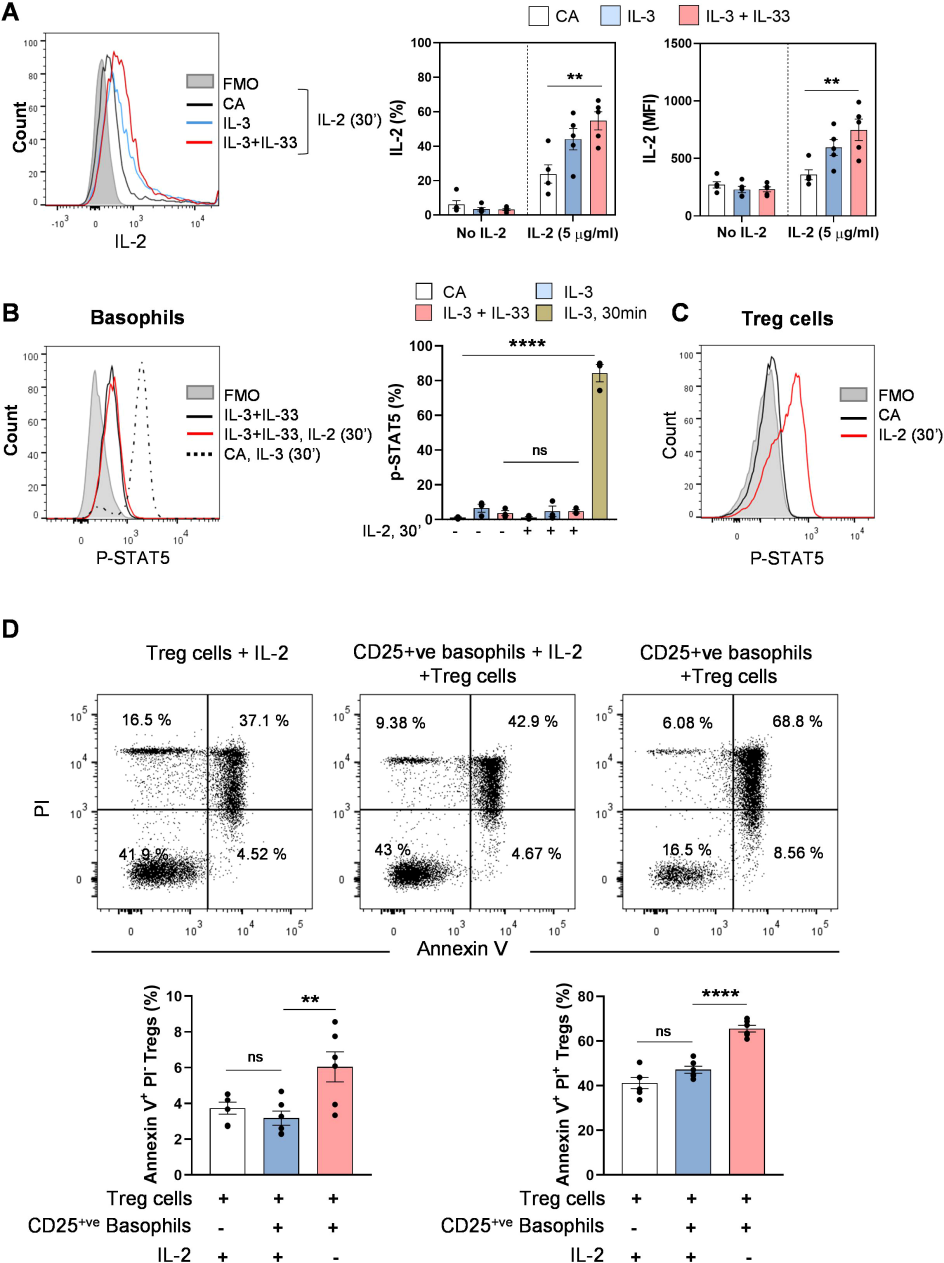
Binding of IL-2 to CD25-expressing basophils neither induces downstream signaling nor limits IL-2 availability to Treg cells. Isolated basophils were cultured alone or in the presence of IL-3 and IL-33 for 24 hrs to induce the expression of CD25. **(A)** Cultured basophils were treated with 5 μg/mL biotinylated-IL-2 for 30 min and stained with PE-streptavidin to detect extracellular IL-2 binding to CD25. Representative FACS histograms and summarized data for binding of IL-2 to basophils (% positive cells and MFI, n = 4). **(B)** Cultured basophils were treated with IL-2 (3000 U/ml) for 30 min, followed by intracellular staining for phosphorylated STAT5 (pSTAT5). IL-3 (10 ng/ml) was added for 30 min on untreated basophils as a positive control for STAT5 activation on basophils. Representative FACS histograms and summarized data showing pSTAT5 expression (% positive cells, n = 3). **(C)** Representative histogram showing the p-STAT5 expression in isolated regulatory T cells following stimulation with IL-2 (3000 U/ml). **(D)** Basophils were treated with IL-3 and IL-33 for 72 hrs, and IL-2 (20 U/ml) was added during last 24 hrs. Subsequently, Treg cells were cocultured with these basophils at a 1:1 ratio, and the viability of Treg cells was analyzed by Annexin-V and PI staining after 48 hrs. Representative dot plots and summarized data from different donors are presented (n = 6). Data are represented as mean ± SEM. *P < 0.05, **P < 0.01, ****P < 0.0001; ns, not significant by One-way ANOVA followed by Tukey’s multiple comparison test. Abbreviation: CA, cells alone; MFI, median fluorescence intensity; PI, propidium iodide.

We then investigated the functional relevance of IL-33-induced CD25 expression on basophils. We have confirmed the ability of CD25-expressing basophils to bind IL-2 cytokine compared to untreated basophils using biotinylated IL-2 (**Figure 3A**). To assess STAT5 activation downstream of the IL-2 receptor, basophils were treated with IL-3 and IL-33 for 24 hrs to induce CD25, washed to remove excess cytokines, and exposed to recombinant IL-2 for 30 min. However, the binding of IL-2 did not induce STAT5 phosphorylation in basophils as determined by intracellular staining for phospho-STAT5 and flow cytometry analysis (**Figure 3B**). On the other hand, treatment of basophils with IL-3 for 30 min significantly induced STAT5 activation via IL-3 receptor signaling, as previously described (21). We have also confirmed the ability of recombinant IL-2 cytokine to induce STAT5 phosphorylation in Treg cells, as an additional positive control (**Figure 3C**).

It is known that functional intermediate (IL-2Rβ/γ_c_ complex) and high-affinity (IL-2Rαβ/γ_c_ complex) IL-2 receptors, upon binding to the IL-2 cytokine, activate Janus kinases and induce STAT5 phosphorylation (22). As functional intermediate or high-affinity IL-2 receptors are minimally expressed on IL-33-stimulated basophils, this explicates the inability of IL-2 to induce canonical downstream signaling in these basophils. We also examined the effect of various doses of IL-2 on the viability of basophils by Annexin V-PI staining. Consistent with the STAT5 phosphorylation data, only IL-3 cytokine significantly decreased apoptosis of basophils, whereas the addition of IL-2 did not produce any further changes in viability (**Supplementary Figure S3**).

Because IL-2 binding to sCD25 has been reported to exert context-dependent effects—ranging from immune activation to suppression (23), we tested whether IL-2 bound to basophil-surface CD25 or sCD25 might influence IL-2 availability for Treg cells, which rely critically on IL-2 for survival. Basophils were stimulated with IL-3 and IL-33 for 72 hrs to induce maximal CD25 expression on both the cell surface and in supernatants, and IL-2 was added at a limiting concentration during the final 24 hours. However, when Treg cells were co-cultured with these CD25-expressing basophils for 48 hrs, their viability remained comparable to that of Treg cells cultured with IL-2 alone (**Figure 3D**). This indicates that, despite the presence of basophil-derived CD25, IL-2 remained accessible to Treg cells.

### 3.4 Basophils from severe COVID-19 patients display enhanced expression of *IL2RA* and *IL2RG*

**Figure 4:**
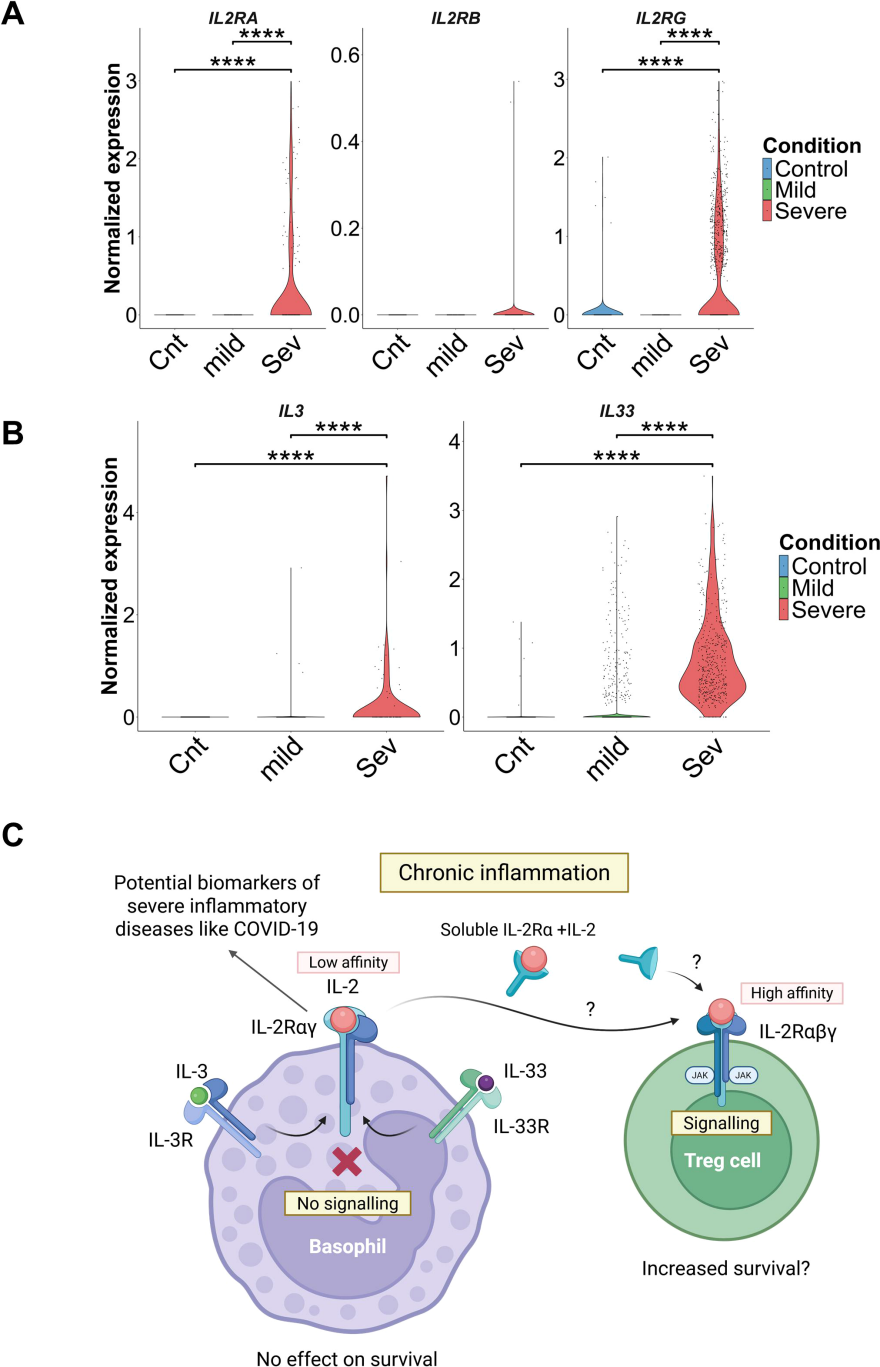
Basophils from severe COVID-19 patients display enhanced expression of *IL2RA* and *IL2RG*. **(A)** Expression of *IL2* receptor subunit transcripts in BALF basophils of healthy controls, mild, and severe COVID-19 patients. Violin plots showing the normalized expression levels of *IL2RA*, *IL2RB*, and *IL2RG* in BALF basophils from healthy controls (Cnt, blue), mild (mild, green), and severe (Sev, red) COVID-19 patients. Individual dots indicate single-cell expression values **(B)** Global Expression of *IL3* and *IL33* in cells from BALF of healthy controls, mild, and severe COVID-19 patients. Violin plot showing the normalized expression of *IL3* and *IL33* in BALF cells from healthy controls (Cnt, blue), mild (mild, green), and severe (Sev, red) COVID-19 patients. Individual dots indicate single-cell expression values. Statistical significance was assessed using Wilcoxon rank-sum test; ****P < 0.0001, *P < 0.05. **(C)** Graphical abstract. IL-3 and IL-33 cytokines synergistically induce CD25 expression in basophils. Transient IL-2 sequestration by CD25^+^ basophils or basophil-derived soluble CD25 could increase the bioavailability of low-dose IL-2 for Treg cells and thereby have a positive impact on their survival. On the other hand, the expression of CD25 and CD132 on basophils might serve as potential biomarkers of severe inflammation like COVID-19. Figure created at BioRender.com.

Previous studies have demonstrated that basophils from allergic asthma patients exhibit increased expression of CD25 (8). We therefore examined whether this enhanced expression of CD25 on basophils is restricted to allergic conditions or represents a broader feature of inflammatory responses. To address this, we analyzed single-cell transcriptomic datasets from mild and severe COVID-19 patients. Consistent with our findings, basophils from severe, but not mild, COVID-19 patients or healthy controls showed a marked upregulation of *IL2RA* (CD25) (*p* < 0.0001). Similarly, the expression of *IL2RG* (CD132) was significantly elevated in severe COVID-19 patients (*p* < 0.001), whereas *IL2RB* (CD122) expression remained largely unchanged across the groups (**Figure 4A**).

Given that IL-33 was responsible for the enhanced expression of CD25 and CD132 on IL-3–primed basophils in our *in-vitro* experiments, we next evaluated the expression of *IL3* and *IL33* in BALF cells from COVID-19 patients. As shown in **Figure 4B**, *IL3* expression was significantly higher in patients with severe COVID-19 compared to both healthy controls and mild cases. Likewise, severe COVID-19 patients exhibited markedly increased *IL33* expression, whereas its expression was negligible in controls and mild cases (**Figure 4B**). We have also analyzed the proportion of Treg cells in control, mild, and severe cases of COVID-19 and found no significant difference between COVID-19 patients and controls (**Supplementary Figure S4**).

Together, these findings suggest that IL3 and IL33 contribute to the upregulation of *IL2RA* and *IL2RG* on basophils in severe COVID-19. Thus, the expression of CD25 and CD132 on basophils might serve as potential biomarkers of severe inflammation like COVID-19.

## 4 Discussion

As a key receptor expressed by various innate and adaptive immune cells, the CD25 receptor has emerged as a disease biomarker and therapeutic target in diseases such as cancer, autoimmunity, and allergy. It is well established that CD25^+^ Treg cells play a crucial role in promoting tolerance to allergens and preventing allergic diseases. Besides, a disrupted balance between CD25^+^ Treg cells and Th2 effector T cells determines allergic disease severity and chronicity. Beyond T cells, recent evidence points to complex and context-dependent roles of CD25 receptor expression on other immune cell types in allergic immune responses (24). In this study, we investigated the regulation of basophil CD25 expression by the alarmin cytokine IL-33 and its functional and physiological implications.

IL-33-mediated activation and accumulation of basophils play crucial roles in the progression of various type 2 allergic and inflammatory diseases through the production of Th2 cytokines (25, 26). Our data revealed that IL-33 uniquely upregulates CD25 expression on IL-3-primed basophils, without further enhancing classical activation markers beyond IL-3’s effects. Mechanistically, IL-3 cytokine signals via activating the JAK/STAT pathway, particularly inducing STAT3 and STAT5 phosphorylation. In contrast, IL-33 triggers a MyD88-dependent signaling cascade resulting in downstream NF-KB and MAP kinase pathway activation, which culminates in nuclear translocation of transcription factors NF-κB and AP-1. Notably, the major transcription factors regulating CD25 transcription include STAT5A, STAT5B, STAT3, NFAT, NF-κB, and AP-1 (27, 28), explaining the synergistic effect of these cytokines on CD25 upregulation. IL-3 exerts a plethora of functions on basophils, including promotion of their survival, activation, priming the degranulation, and extravasation (29–32).

Prior studies also reported that IL-33 induces CD25 expression in ILC2 and Treg cells, and the interaction between these two cell types is important for Treg maintenance and survival (33). Moreover, IL-33-treated mice exhibit high frequencies of CD25-expressing B cells with an unconventional Breg-like phenotype. These cells can suppress effector T cell expansion, and the adoptive transfer of these B cells can block the development of spontaneous colitis in IL-10^−/−^ mice (34). These findings underscore the multifaceted role of IL-33 in modulating CD25 expression across diverse immune cells, shaping the immune landscape in type 2 inflammation and allergic diseases.

The low expression of CD122 (IL-2Rβ) subunit on stimulated basophils accounts for the lack of downstream signaling effects, such as STAT5 phosphorylation or survival effects, which we have observed in response to IL-2. Similar phenomena are described in activated dendritic cells (DCs), which express CD25 without CD122, and therefore remain intrinsically unresponsive to IL-2 signaling (35, 36). Although basophil-derived CD25 can bind IL-2, our co-culture experiments indicate that this binding is reversible in the presence of Treg cells, which express the high-affinity trimeric IL-2 receptor. Consequently, IL-2 availability to Treg cells remains sufficient, and their viability is unaffected. Various studies have reported that CD25-expressing cells or sCD25 can modulate IL-2-responsive cells either positively or negatively, although the data remain controversial. von Bergwelt-Baildon et al. have previously reported that the maturation of DCs in the presence of prostaglandin (PG)-E2 induces the expression and secretion of CD25 and inhibits T cell proliferation, likely due to IL-2 capture by soluble CD25 (37). In contrast, CD25-expressing DCs can trans-present IL-2 to T cells in an antigen-specific manner to facilitate early high-affinity signaling and promote subsequent T cell expansion (38).

Although basophils are reported to have antigen presentation functions under specific experimental conditions in mice (39), human basophils lack the features of professional APCs, including HLA-DR expression, and they primarily modulate T cell responses through soluble mediators or cooperating with other APCs (40, 41). A recent study by Nickle et al. proposes that sCD25 can reversibly sequester IL-2, limiting acute maximal proliferative responses while preserving IL-2 bioavailability to maintain low-zone IL-2 signaling and support the preferential expansion of CD25^high^ memory and Treg subsets (42). Taken together, although CD25^+^ basophils and basophil-derived sCD25 may function as IL-2 “sinks,” their biological consequences are likely context-dependent and warrant further detailed investigation.

IL-3 and IL-33 cytokines have crucial roles in orchestrating immune cell activation and shaping disease progression and tissue damage in severe inflammatory conditions such as asthma and COVID-19 (43, 44). SARS-CoV-2 infection triggers the release of multiple inflammatory cytokines through signaling pathways activated by pattern recognition receptors. In severe COVID-19, elevated IL-3 and IL-33 in the lung microenvironment suggest a shift toward innate “alarmin” driven, type-2 inflammation. IL-33 is an epithelial alarmin whose serum and BALF levels correlate strongly with disease severity (45). In fact, IL-3 dramatically boosts SARS–CoV–2–induced basophil IL-13 and IL-4 secretion (46). Thus, our finding of concurrent *IL3* and *IL33* upregulation in BALF cells is concordant with reports that SARS-CoV-2 infection triggers IL-33 release from damaged epithelium (initiating a type-2 response) (45).

Notably, we also observed marked upregulation of *IL2RA* and *IL2RG* on basophils in severe COVID-19 patients. In clinical contexts, CD25 is used as one of the minor diagnostic markers to discriminate neoplastic mast cells from normal/reactive mast cells in indolent mast cell disease, and CD25 expression indicates histologically occult bone marrow infiltration and residual disease after therapy (47, 48). More recently, CD25 has been reported as a unique marker on blood basophils in patients with stable-mildly symptomatic forms of allergic asthma while, the expression of other classical basophil activation/degranulation markers, such as CD69, CD203, and CD63, remained comparable to healthy individuals (8), indicating the predictive potential of CD25 expression on basophils as a biomarker of disease prognosis and treatment efficacy in asthma. Our data highlight that CD25 expression on basophils could also be a potential biomarker in severe COVID-19 patients, wherein a subset of severe patients had significantly enhanced transcripts of *IL2RA*.

Several studies have reported persistent or increased Treg cell frequencies in severe COVID-19 cases (49–51). Even in settings where IL-2 is limited, cytokines such as IL-7 and IL-15 can compensate to support Treg survival and proliferation, particularly under inflammatory conditions (52). Additionally, tissue damage can lead to the release of TGF-β, which promotes the differentiation of naïve T cells into inducible Treg (iTreg) cells. IL-33 released from injured lung epithelium can further enhance Treg activity by inducing the production of IL-3 and IL-13, thereby suppressing inflammatory cytokine responses. Thus, intrinsic feedback mechanisms during systemic inflammation generally favor Treg maintenance or expansion. These dynamics have important implications: elevated Treg frequencies may help limit immune-mediated tissue damage and cytokine storms, but could also impair pathogen clearance. Conversely, some studies have reported decreased Treg frequencies in COVID-19 patients (53). During acute inflammation, pro-inflammatory cytokines such as IL-6 and TNF-α can destabilize FoxP3 expression, causing a transient decline in Treg numbers (54). Nevertheless, the majority of evidence suggests that strong systemic inflammation promotes Treg persistence and expansion, consistent with our analysis. Whether transient IL-2 sequestration and release by basophil-derived CD25 contributes to maintaining Treg populations during inflammation remains an open question.

In conclusion, our data demonstrate that IL-33 cytokine promotes CD25 expression in primed human basophils. Higher levels of IL-3 and IL-33 cytokines in inflammatory environments explain the higher levels of CD25 expression in allergic patients, as well as in severe COVID-19 patients. However, we found that the CD25-expressing basophils are not responsive to IL-2 despite their ability to bind IL-2. While CD25^+^ basophils and basophil-derived sCD25 have the potential to function as IL-2 “sinks,” IL-2 remains accessible to Treg cells, enhancing their viability (**Figure 4C**). The potential physiological impacts of this IL-2 capture by basophils warrant further investigation.

## Data Availability

The original contributions presented in the study are included in the article/**Supplementary Material**. Further inquiries can be directed to the corresponding author.
