# Supplementary material for: IL-33 and IL-3 synergistically induce CD25 expression on human basophils without functional IL-2 signaling: a potential marker of severe COVID-19
Source: Front Immunol. 2025 Dec 11;16:1718240. doi: 10.3389/fimmu.2025.1718240 (PMC12738815; doi:10.3389/fimmu.2025.1718240)
Supplement: Supplementary file 1 [file DataSheet1.pdf]

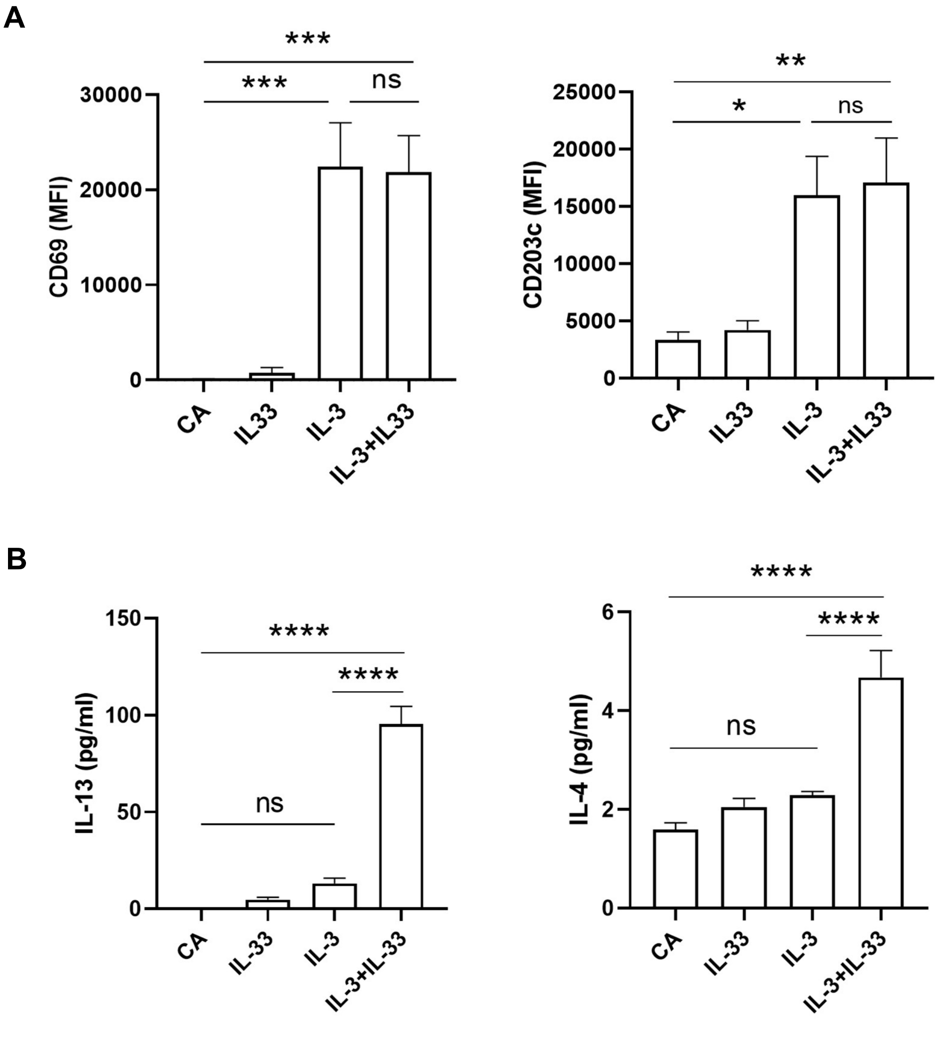

Suppl. Fig 1. A. Expression of basophil activation markers CD69 and CD203c on basophils treated with IL-3 or IL-33 for 24 hrs (MFI, n = 6). **B**. Amount of Th2 cytokines (IL-13 and IL-4) in the culture supernatants of stimulated basophils measured by ELISA (n=8). Data are represented as mean ± SEM. *$P < 0.05$, **$P < 0.01$, ns, not significant by one-way ANOVA with Tukey's multiple comparison test. Abbreviation: CA, cells alone; MFI, median fluorescence intensity.

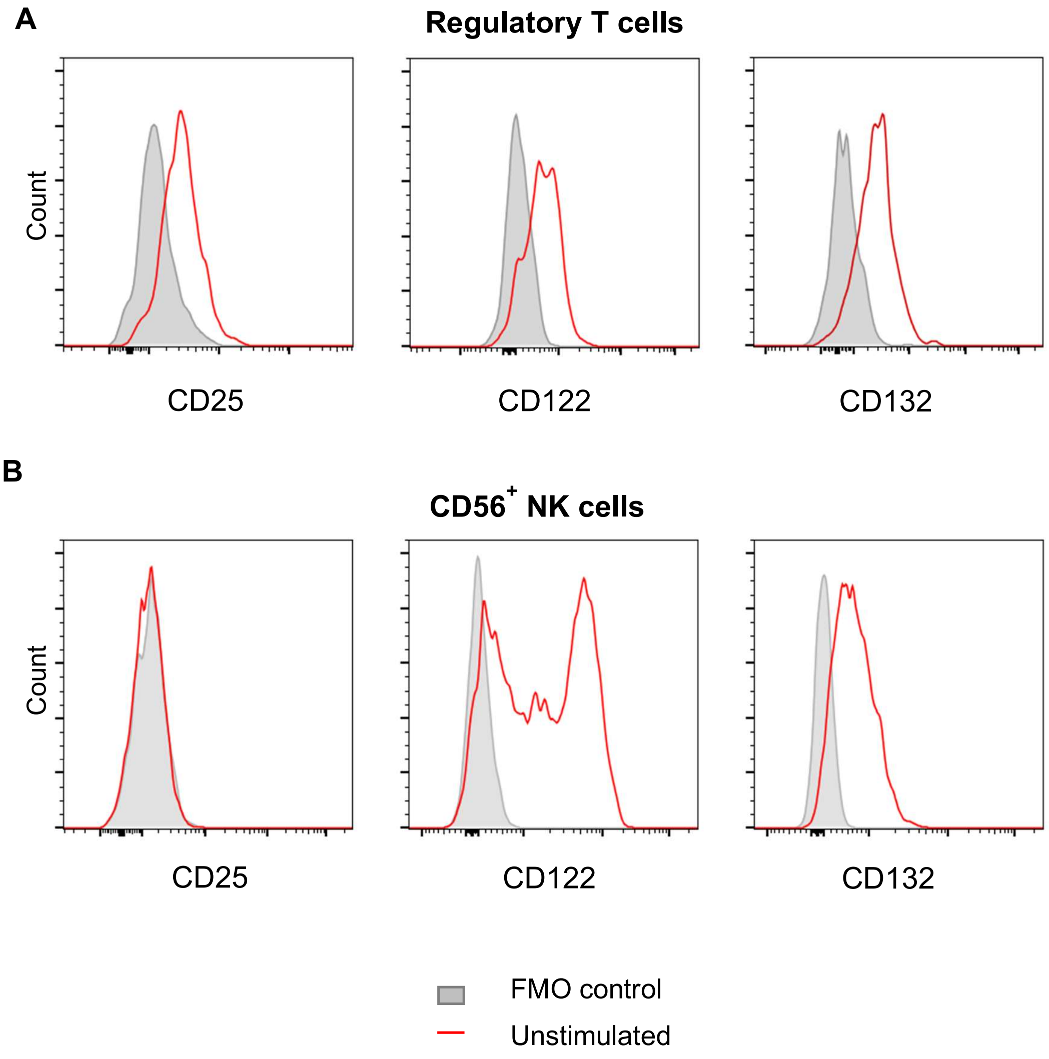

Suppl. Fig 2. Representative histograms showing the expression of CD25, CD122 and CD132 in **(A)** isolated Treg cells and **(B)** CD56^{+} NK cells.

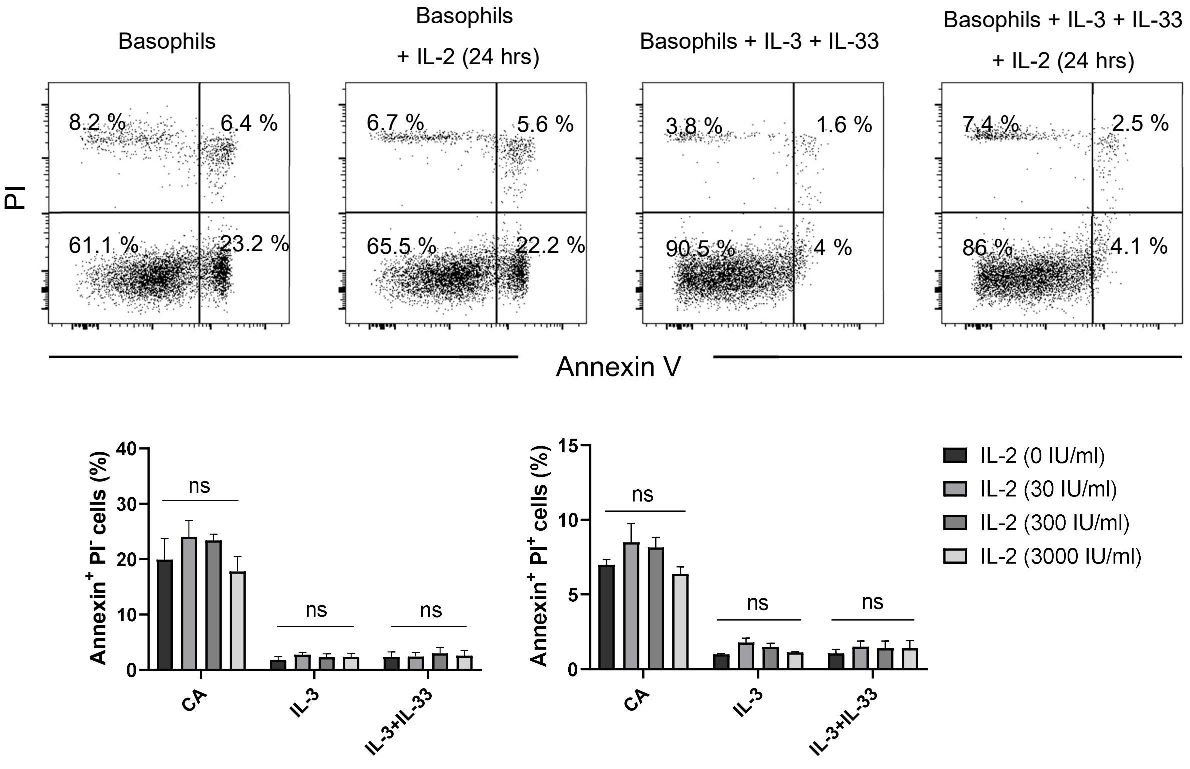

Suppl. Fig 3. The viability of basophils analyzed by Annexin-V and PI staining after treatment with the indicated cytokines for 24 hrs. Representative dot plots and summarized data from different donors are presented (mean ± SEM, n = 3). **P < 0.05*; ns, not significant by One-way ANOVA followed by Tukey's multiple comparison test. Abbreviation: CA, cells alone; MFI, median fluorescence intensity.

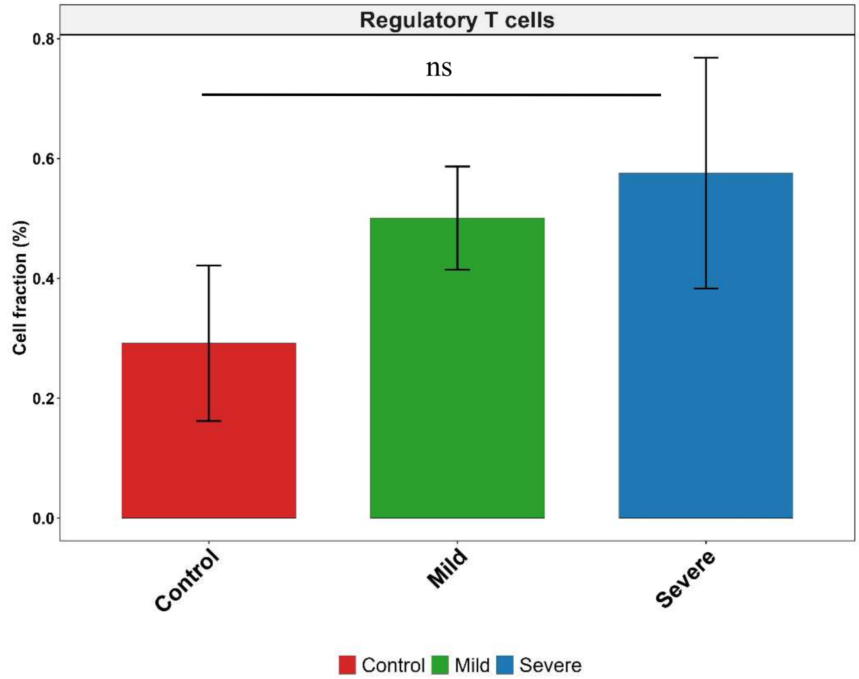

Suppl. Fig 4: Box plot showing the proportion of regulatory T cells (%) in BALF of healthy controls, mild, and severe COVID-19 patients. ns, not significant by One-way ANOVA followed by Tukey's multiple comparison test.
